# Prophylactic cranial irradiation in small cell lung cancer: A review of evidence

**DOI:** 10.7555/JBR.38.20240293

**Published:** 2025-01-07

**Authors:** Femi Williams Adeoye

**Affiliations:** 1 Oncology Department, Southend University Hospital, Mid and South Essex NHS Foundation Trust, Southend-on-Sea, Essex SS0 0RY, UK; 2 The Institute of Cancer Research, London SW7 3RP, UK

Small cell lung cancer (SCLC), which accounts for about one-sixth of all lung cancer cases, is the most aggressive subtype, with a high propensity for brain involvement^[1]^. The role of prophylactic cranial irradiation (PCI) in reducing intracranial relapse and improving survival has been a subject of intense debate for decades. Although earlier trials demonstrated its benefits in both early and advanced stages of the disease, the advent of more advanced imaging modalities, the availability of newer systemic anti-cancer therapies, and significant improvement in radiotherapy techniques have made this practice increasingly controversial within the oncology community. This article reviews the existing evidence in light of these recent developments and explores perspectives on the future of PCI in SCLC.

There are different staging systems for SCLC, but the broad classification into limited-stage (LS) and extensive-stage (ES) diseases will be used in this article, as is commonly done in clinical trials. According to the American Joint Committee on Cancer-defined tumor-node-metastasis staging system, ES-SCLC includes any T, any N, M1a–c, and T3–4 tumors because of multiple lung nodule involvement. LS-SCLC includes any T, any N, and M0 (except for T3–4) when multiple lung nodules do not fit within a single radiation field^[2]^. While approximately 10%–25% of SCLC patients are diagnosed with brain metastases (BM) at initial presentation, an additional 40%–50% will develop intracranial relapse over the course of their diseases^[1]^. In addition to the high propensity for brain involvement in SCLC, there is limited penetration of the blood-brain barrier by the routinely used chemotherapeutic agents. Consequently, intracranial relapse often leads to death in patients who otherwise have no other sites of disease activity. This growing recognition that the brain may serve as a sanctuary site for microscopic disease has led to investigation into the benefit of PCI. Following the results of earlier trials, PCI was initially adopted as the standard of care for LS-SCLC and was later extended to ES-SCLC.

In LS-SCLC, PCI has been shown to significantly reduce the risk of symptomatic BM and improve overall survival (OS). A landmark meta-analysis, which analyzed data from over 987 patients in complete remission across seven trials, demonstrated both progression-free survival (PFS) and OS benefits. PCI was associated with a significant reduction in the cumulative incidence of BM (relative risk [RR] = 0.46; 95% confidence interval [CI]: 0.38–0.57; *P* < 0.01). Additionally, the RR of death in the PCI group was 0.84 (95% CI: 0.73–0.97), corresponding to a 5.4% survival benefit at three years^[3]^.

Whilst these findings are compelling, the study has some limitations that raise concerns about its applicability in modern clinical practice. A major concern is the heterogeneity in the design of the included studies. Additionally, the imaging modalities used for staging (*i.e.*, chest radiography and CT scan) are significantly inferior to contemporary techniques such as positron emission tomography (PET) and magnetic resonance imaging (MRI) scans. Therefore, many patients originally classified as having LS-SCLC in these studies would likely be upstaged to ES-SCLC with the use of modern imaging techniques. Moreover, autopsy studies have reported the central nervous system (CNS) involvement in approximately 65% of SCLC patients, with the probability approaching 80% in patients who survived for two years or longer^[4]^. There is a strong possibility that a significant proportion of patients deemed disease-free at recruitment or follow-up actually had undetected BM, raising the question of whether the benefits of PCI demonstrated in the meta-analysis were truly therapeutic. Additionally, the study did not address concerns about the neurocognitive sequelae of PCI, a critical factor when evaluating its role in clinical practice.

Against this backdrop, a recent systematic review and meta-analysis was conducted to evaluate the benefit of PCI in SCLC with radiologically confirmed the absence of BM after the first-line treatment. Although the study demonstrated an OS benefit across all stages (hazards ratio [HR] = 0.59; 95% CI: 0.55–0.63; *P* < 0.001), subgroup analyses of studies using MRI showed no significant OS difference in patients with LS-SCLC regardless of PCI status (HR = 0.74; 95% CI: 0.52–1.05; *P* = 0.08)^[5]^. This further reinforces the notion that the survival benefits identified in earlier studies might be therapeutic rather than prophylactic. Interestingly, a retrospective study investigating the survival benefits of PCI enrolled 500 patients with LS-SCLC who were followed up with brain MRI (preferably) or enhanced CT or PET scans after definitive chemoradiotherapy^[6]^. The 12-month and 36-month OS rates in the PCI group were 87.5% and 42.3%, respectively, compared with 70.4% and 20.9% in the non-PCI group (*P* = 0.002). Although these findings support the use of PCI in LS-SCLC, it is important to note that, aside from its retrospective design, a key limitation of this study was that the frequency of follow-up imaging was left to the discretion of the physicians.

There are several noteworthy limitations of PCI in this context. Firstly, PCI has not been shown to reduce the risk of intrathoracic or distant extracranial recurrence^[3]^. Secondly, a retrospective review of 658 patients with LS-SCLC who underwent PCI at MD Anderson Cancer Centre found no survival advantage in patients older than 70 years^[7]^. Additionally, a separate retrospective study conducted at the Shanghai Chest Hospital observed a relatively lower risk of BM in patients with resected stage Ⅰ disease, suggesting a diminished efficacy of PCI in this group^[8]^. Therefore, there should be less emphasis on recommending PCI for elderly patients with multiple comorbidities or those with resected stage Ⅰ disease.

The benefit of PCI is more debatable in ES-SCLC, which accounts for approximately two-thirds of all SCLC cases. In 2007, the European Organisation for Research and Treatment of Cancer (EORTC) published the results of a phase Ⅲ study investigating the efficacy of PCI in ES-SCLC. The trial recruited 286 patients with ES-SCLC, who were randomly assigned to either the PCI or observational arm. The one-year cumulative risk of symptomatic BM was 14.6% in the PCI arm compared with 40.4% in the observational arm, while the 12-month survival was 27.1% in the PCI arm versus 13.3% in the control arm^[9]^. These findings demonstrated that PCI reduced the risk of symptomatic BM and provided an OS benefit, leading to its increased adoption in the management of ES-SCLC following the publication of this study.

However, this study also has several limitations. Firstly, the dose fractionation scheduled for PCI varied substantially, which may have affected the consistency of results. Secondly, brain imaging was not mandated unless patients developed clinical symptoms suggestive of BM. As a result, the study may have inadvertently enrolled patients with subclinical BM. Therefore, the longer OS observed in patients who received PCI may have reflected the responses of pre-existing intracranial lesions to radiotherapy rather than the preventive benefits of PCI.

Another challenge in applying the EORTC approach in clinical practice is the objective of the study, which was to assess the benefit of PCI in reducing symptomatic BM. Patients with symptomatic BM are more likely to have a higher intracranial disease burden of the disease, making them less suitable for surgical resection or stereotactic radiosurgery and more likely to experience poorer outcomes^[10]^. In modern clinical practice, the ideal approach would be to detect intracranial lesions at an earlier stage.

More recently, a multicenter Japanese study recruited 224 patients with ES-SCLC, all of whom had responded to initial chemotherapy and had no MRI evidence of intracranial disease, to evaluate the benefit of PCI. Subjects were randomly assigned (1∶1) to receive PCI (25 Gy/10 fractions) or observation alone. All ES-SCLC patients were required to undergo brain MRI scans every three months for the first 12 months as well as at 18 and 24 months. This study was discontinued early because of futility, as no significant OS or PFS benefit was observed. In the final analysis, the median OS was 11.6 months (95% CI: 9.5–13.3) in the PCI group and 13.7 months (95% CI: 10.2–16.4) in the control arm (HR = 1.27; 95% CI: 0.96–1.68; *P* = 0.094)^[11]^. These results challenged the survival advantage observed in the earlier EORTC study^[9]^_._

The contrasting results from these two studies make it difficult to strongly advocate for PCI in this group. The conflicting findings may be partly due to fundamental differences in the study designs. The Japanese study recruited only patients with no MRI evidence of the disease, while routine brain imaging was not mandated in the EORTC study. The EORTC recommendation may be a pragmatic approach in centers where MRI facilities are not readily available. It is also a reasonable option for patients who have had an excellent response to initial chemo-radiotherapy and a confirmed absence of BM to decline PCI, if they can be followed up with periodic brain MRI.

**PCI in the current era of immunotherapy:** While there have been no major advancements in chemotherapeutic options for the first and subsequent lines of treatments in recent decades, immune checkpoint inhibitors have recently emerged as a promising addition to the treatment landscape. The IMpower133 trial demonstrated both PFS and OS benefits with atezolizumab, an anti-programmed death-ligand 1 (anti-PD-L1) agent, when combined with chemotherapy in patients with ES-SCLC^[12]^. As a result, atezolizumab has become a standard treatment option for this patient population. In the maintenance phase of the IMpower133 trial, 22 subjects in each arm received PCI, but the results showed no difference in OS and PFS between the two arms. However, the study was not statistically powered to determine the non-inferiority of omitting PCI. Interestingly, the Atezo-Brain study, a recent phase Ⅱ trial, suggested that atezolizumab might have efficacy in patients with non-small cell lung cancer (NSCLC) and untreated BM^[13]^. Similarly, the PACIFIC trial, which evaluated the benefits of adjuvant durvalumab (another anti-PD-L1 agent) in unresectable NSCLC, demonstrated a reduced risk of BM^[14]^.

Although the CASPIAN trial also demonstrated an OS benefit of durvalumab in ES-SCLC (HR = 0.73; 95% CI: 0.59–0.91; *P* = 0.0047)^[15]^, as seen in the Impower133 trial^[12]^, only a limited number of participants (< 10%) had PCI, because it was only administered at discretion of the investigators. Therefore, no definitive conclusions can be drawn about its benefit in this context. The improved penetration of the blood-brain barrier by immune checkpoint inhibitors may offer two insights. On one hand, the response observed in patients with intracranial disease and reduction in central nervous system relapse risks may attenuate the previously observed benefits of PCI, therefore obviating the need for radiotherapy in the future. On the other hand, radiotherapy may enhance the effect of immunotherapy, leading to better response and improved outcomes with combined modalities. However, more research is needed to determine the optimal approach for integrating these treatment strategies.

Overall, the emergence of newer and more effective systemic anticancer therapies with improved CNS penetration may lead to a global decline in the use of PCI in the future. The ongoing PRIMALung study^[16]^, an international phase Ⅲ trial sponsored by EORTC, holds potential to provide critical insight into this evolving treatment paradigm.

**Toxicities and dose fractionation:** Early and late side effects of PCI remain a key challenge. In the Japanese trial^[11]^, PCI was associated with a slightly higher degree of acute toxicities, but there was no difference in cognitive function. The EORTC trial^[9]^ showed that PCI was associated with an increased severity of fatigue, anorexia, and lower limb weakness during the first three months. A major concern with PCI has been its potential neurotoxicities. The recent adoption of advanced radiotherapy techniques, such as volumetric modulated arc therapy and intensity-modulated radiotherapy, allows sparing of the peri-hippocampal areas responsible for memory, learning, and spatial navigation. Although hippocampal sparing has been considered neuroprotective, a recent hippocampal avoidance-PCI trial showed that while it reduced the risk of hippocampal atrophy at 12 months (3.0% versus 5.8%), it did not translate into a reduced neurocognitive decline^[17]^. Another challenge with hippocampal avoidance is the concern about the potential risk of relapse in the spared peri-hippocampal areas.

Interestingly, a prospective study that conducted neurocognitive assessments before and after PCI in 69 patients with SCLC found evidence of impaired cognitive function in 47% of patients before PCI. Univariate analysis showed a significant but transient decline in language and executive functions following PCI. However, after adjusting for non-CNS disease, the deficit in the executive functional domain was no longer statistically significant^[18]^. Therefore, neurocognitive changes in this population might be partly explained by factors such as paraneoplastic syndromes, subclinical microscopic CNS disease, or "chemo brain", rather than being solely caused by irradiation^[3,19]^.

The optimal dose fractionation for PCI remained uncertain for years, with the commonly used schedules ranging from 36 Gy in 18 fractions to 20 Gy in five fractions. Overall, fractionation studies have shown a trend favoring the 25 Gy in 10 fractions regimen, which was associated with fewer side effects, improved survival outcomes, and a non-inferior intracranial relapse rate, compared with higher-dose regimens^[20]^.

In conclusion, while evidence supports the potential benefits of PCI in both LS- and ES-SCLC, maintaining patients' quality of life remains a critical consideration. The benefits and drawbacks of PCI should be discussed with patients to promote shared decision-making. The decision to offer PCI should be individualized, taking into account each patient's risk profile, preferences, and available resources.

Looking ahead, it is anticipated that more well-designed prospective RCTs conducted in the current era, characterized by advanced, improved radiotherapy techniques, and the growing integration of immunotherapy, will provide valuable new insights into the role of PCI in SCLC management.
